# Impact of Trump's Promotion of Unproven COVID-19 Treatments and Subsequent Internet Trends: Observational Study

**DOI:** 10.2196/20044

**Published:** 2020-11-20

**Authors:** Kacper Niburski, Oskar Niburski

**Affiliations:** 1 McGill University Montreal, QC Canada; 2 York University Toronto, ON Canada

**Keywords:** COVID-19, behavioral economics, public health, behavior, economics, media, influence, infodemic, infodemiology, infoveillance, Twitter, analysis, trend

## Abstract

**Background:**

Individuals with large followings can influence public opinions and behaviors, especially during a pandemic. In the early days of the pandemic, US president Donald J Trump has endorsed the use of unproven therapies. Subsequently, a death attributed to the wrongful ingestion of a chloroquine-containing compound occurred.

**Objective:**

We investigated Donald J Trump’s speeches and Twitter posts, as well as Google searches and Amazon purchases, and television airtime for mentions of hydroxychloroquine, chloroquine, azithromycin, and remdesivir.

**Methods:**

Twitter sourcing was catalogued with Factba.se, and analytics data, both past and present, were analyzed with Tweet Binder to assess average analytics data on key metrics. Donald J Trump’s time spent discussing unverified treatments on the United States’ 5 largest TV stations was catalogued with the Global Database of Events, Language, and Tone, and his speech transcripts were obtained from White House briefings. Google searches and shopping trends were analyzed with Google Trends. Amazon purchases were assessed using Helium 10 software.

**Results:**

From March 1 to April 30, 2020, Donald J Trump made 11 tweets about unproven therapies and mentioned these therapies 65 times in White House briefings, especially touting hydroxychloroquine and chloroquine. These tweets had an impression reach of 300% above Donald J Trump’s average. Following these tweets, at least 2% of airtime on conservative networks for treatment modalities like azithromycin and continuous mentions of such treatments were observed on stations like Fox News. Google searches and purchases increased following his first press conference on March 19, 2020, and increased again following his tweets on March 21, 2020. The same is true for medications on Amazon, with purchases for medicine substitutes, such as hydroxychloroquine, increasing by 200%.

**Conclusions:**

Individuals in positions of power can sway public purchasing, resulting in undesired effects when the individuals’ claims are unverified. Public health officials must work to dissuade the use of unproven treatments for COVID-19.

## Introduction

Numerous treatments have been suggested for the novel COVID-19 disease, with only remdesivir recently showing some potential efficacy prior to its approval on May 1, 2020 [1]. The efficacy of other therapies, such as hydroxychloroquine, chloroquine, and azithromycin, remains unproven, though they have been praised by numerous public figures, such as US president Donald J Trump.

Previous studies have shown that individuals with high influence (ie, individuals with large social capital or political power) [2] can affect public decisions and purchasing power [3]. Moreover, many of these individuals have the ability to affect content, sentiment, and public attention by using social media to disseminate information [4]. Donald J Trump is one such individual who has previously influenced behavior through his high social presence on the internet and his political capital as the president of the United States [5].

In 2020, during the initial period of the COVID-19 pandemic, many people were focused on uninvestigated therapies, especially Donald J Trump. However, it has become increasingly clear that some therapies, such as hydroxychloroquine, had numerous concerns surrounding them. Studies have reported that hydroxychloroquine can cause conduction disturbances and fatal arrhythmias [6], the supply of hydroxychloroquine may decrease for approved conditions like rheumatoid arthritis [7], and chloroquine is similar to chloroquine products that are used as aquarium cleaners, which may be toxic if ingested in large quantities [8]. A death has already occurred due to the ingestion of chloroquine products [9].

A recent study has noted that there was an influx of internet searches for hydroxychloroquine and chloroquine after being tweeted about by US president Donald J Trump [10]. However, the study did not investigate all therapies that were initially suggested in the United States, the purchasing amounts on Google and Amazon, or the television airtime for each respective medication. The study also did not associate these purchasing amounts with Donald J Trump’s discussions and tweets, which are 2 avenues where his reach is in the millions. This would have better shown a relationship between his influence and the impact he has on his supporters and the general public.

We therefore investigated the relationship between Donald J Trump’s advocacy for unproven treatments and the media landscape of COVID-19 treatments. We also investigated how these coalesced into behavioral changes in individuals. We analyzed the time Donald J Trump spent discussing hydroxychloroquine, chloroquine, azithromycin, and remdesivir on the 5 largest US television stations, the total number of searches and purchases on Google and Amazon for these treatments, and the Twitter statistics and fallout of Donald J Trump’s advocacy for these therapies.

## Methods

### Twitter

Tweets were catalogued based on mentions of hydroxychloroquine, chloroquine, azithromycin, and remdesivir. Factba.se was used to note if any Twitter posts were archived or otherwise hidden by Donald J Trump [11]. Tweet Binder was used to assess average analytics data on key metrics, such as likes, retweets, and comments [12]. Tweet Binder further aggregated all tweets and averaged longitudinal tweeting patterns.

### Television

The time Donald J Trump spent discussing each treatment on television was recorded from the 5 largest US television stations: CNN (Cable News Network), C-SPAN (Cable-Satellite Public Affairs Network), Fox News, MSNBC (Microsoft/National Broadcasting Company), and BBC (British Broadcasting Corporation) News. The Global Database of Events, Language, and Tone was used to note White House briefings and the total monitored airtime of each station for broadcasts that aired from March 1 to April 30, 2020 [13]. Airtime data are presented as percentages of 15-second airtime blocks, which is the maximum the software can crawl its historical database. Manual crawling of television airtimes or news headings was not performed.

### Google

Google searches from March 1 to April 30, 2020 were indexed with Google Trends, focusing on searches performed within the United States [14]. Searches including the words “hydroxychloroquine,” “chloroquine,” “azithromycin,” “remdesivir,” and “covid treatment” were catalogued, along with the Google purchasing patterns for these items. Analyses for these searches were performed concurrently with cataloguing. However, the results for this period were not large enough to report. The data obtained were the proportional patterns of searches instead of exact amounts, given Google’s data agreement [15].

### Amazon

Amazon purchases were indexed based on the following words: “hydroxychloroquine,” “chloroquine,” “azithromycin,” and “remdesivir.” Search volume was assessed using Helium 10 software [16]. Estimated purchase amounts were calculated using Ahrefs [17].

### Data Analysis

Data were analyzed with Microsoft Excel. This included aggregating the data, cataloguing data, and formatting data with key points, such as Donald J Trump’s first press conference on March 19, 2020 or his numerous tweeting dates.

The wide variety of sampling, numerous software-defined metrics, and methods of measuring outcomes, such as gross estimates and proportions instead of true samples, did not lend themselves to statistical analysis [18]. The key assumption of transitivity, which assumes no important differences in the distribution of either potential effect modifiers or sampling techniques (eg, individual comparisons of price points between Google and Amazon), would be high for indirect comparisons.

## Results

### Twitter

Table 1 notes the characteristics of US president Donald J Trump’s tweets. Donald J Trump mainly focused on hydroxychloroquine, as per his own tweets (eg, his tweets on March 21, 2020) and his retweets of Twitter posts from other people who supported his claims regarding the efficacy of hydroxychloroquine. Various tweets included news articles (eg, the tweet on April 4, 2020), which were mostly from Fox News (4/6, 67%).

Based on our analysis with Tweet Binder [19], with an average proportion of nearly 100,000 likes and 20,000 retweets, Donald J Trump’s first tweet on March 21, 2020 advocating for the use of hydroxychloroquine and azithromycin was one of his most popular tweets. With 385,700 likes and 103,200 retweets at the time of this study, the tweet had a potential estimated impression reach (ie, the number of potential user views on the individual tweet) of 78,800,580.

**Table 1:** Characteristics Donald J Trump’s tweets that mention hydroxychloroquine, chloroquine, chloroquine, and remdesivir, including key metrics such as retweets, likes, and whether the tweets were retweets or self-composed tweets by Donald J Trump.

Therapy/Mention		Date	Tweet sources	Number of retweets of other posts	Number of self-retweets	Number of retweets	Number of likes
**Hydroxychloroquine**							
	1	April 18, 2020	@paulsperry_ [20]	1	0	12,600	30,500
	2	April 10, 2020	@cybergenica [21]	1	0	5800	16,900
	3	April 4, 2020	@OANN [22]	1	0	7800	21,600
	4	April 4, 2020	@jsolomonReports [23]	1	0	11,300	28,200
	5	March 23, 2020	@AndrewCMcCarthy [24]	1	0	9100	23,700
	6	March 21, 2020	@realDonaldTrump [25]	0	1	103,200	385,700
	7	March 21, 2020	@MichaelCoudrey [26]	1	0	27,300	53,900
	8	March 21, 2020	@realDonaldTrump [27]	1	0	103,200	385,700
**Azithromycin**							
	1	March 21, 2020	@realDonaldTrump [25]	0	1	103,200	385,700
	2	March 21, 2020	@MichaelCoudrey [26]	1	0	27,300	53,900
	3	March 21, 2020	@realDonaldTrump [27]	0	0	103,200	385,700
**Chloroquine**							
	0	N/A^a^	0	0	0	0	0
**Remdesivir**							
	0	N/A	0	0	0	0	0

^a^N/A: not applicable.

### Television Mentions and Broadcasts

Table 2 notes the number of times Donald J Trump mentioned COVID-19 treatments during White House briefings. He mentions hydroxychloroquine 37 times in total, most frequently on April 4, 2020 (9 times); chloroquine 12 times in total, most frequently on March 19, 2020 (7 times); azithromycin 8 times in total, most frequently on March 19, 2020 (3 times, while also mentioning hydroxychloroquine); and remdesivir 8 times in total, most frequently on March 19, 2020 (4 times).

**Table 2:** Dates of Donald J Trump’s mentions of unproven therapies during televised appearances.

Date	Number of mentions on television			
	Hydroxychloroquine	Chloroquine	Azithromycin	Remdesivir
March 19, 2020	4	7	3	4
March 21, 2020	3	0	0	1
March 23, 2020	2	0	0	0
March 27, 2020	1	1	1	0
March 29, 2020	1	0	0	0
March 31, 2020	4	2	0	0
April 1, 2020	1	1	0	1
April 4, 2020	9	0	0	0
April 5, 2020	3	0	0	1
April 6, 2020	1	0	0	0
April 7, 2020	3	0	1	0
April 8, 2020	1	0	0	0
April 9, 2020	1	0	1	0
April 10, 2020	2	0	1	0
April 13, 2020	1	1	1	1
Total	37	12	8	8

**Figure 1:**
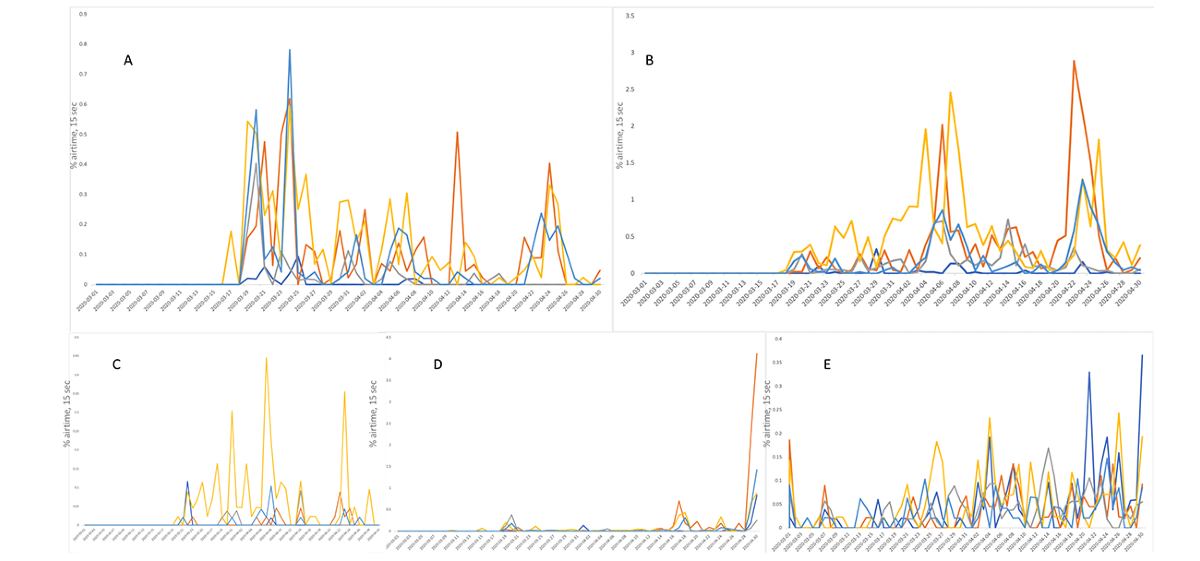
Television airtime of keywords from March 1 to April 30, 2020 during 15-second airtime blocks. Dark blue represents BBC News, orange represents CNN, grey represents C-SPAN, yellow represents Fox News, and light blue represents MSNBC. (A) Airtime for the term "chloroquine". (B) Airtime for the term "hydroxychloroquine." (C) Airtime for the term "azithromycin." (D) Airtime for the term "remdesivir". (E) Airtime for the term "covid treatment". CNN: Cable News Network; C-SPAN: Cable-Satellite Public Affairs Network.

Figure 1 displays the television airtime of broadcasts that mentioned the treatments. Airtime for mentions of chloroquine (Figure 1A) peaked on March 24, 2020 on Fox News (0.59%), MSNBC (0.78%), and CNN (0.61%), following the chloroquine-related death. Following Donald J Trump’s tweet advocating for the use of hydroxychloroquine, airtime for mentions of hydroxychloroquine (Figure 1B) peaked on April 4 and April 6, 2020 on Fox News (1.94% and 2.46%, respectively), while peaking on CNN on April 22, 2020 (2.88%). Fox News was one of the single television networks that continually provided coverage on azithromycin (Figure 1C), as airtime for its mention peaked at 0.45% on April 7, 2020. Remdesivir (Figure 1D) was not mentioned on any news organizations until April 30, 2020, followed by the decision by the US Food and Drug Administration to approve remdesivir as a COVID-19 treatment on May 1, 2020 [28]. At this point, airtime for mentions of remdesivir experienced peaks comparable to those of the other treatments. COVID-19 treatment coverage has been increasing on all networks, especially after the March 19, 2020 press conference.

### Google and Amazon

**Figure 2:**
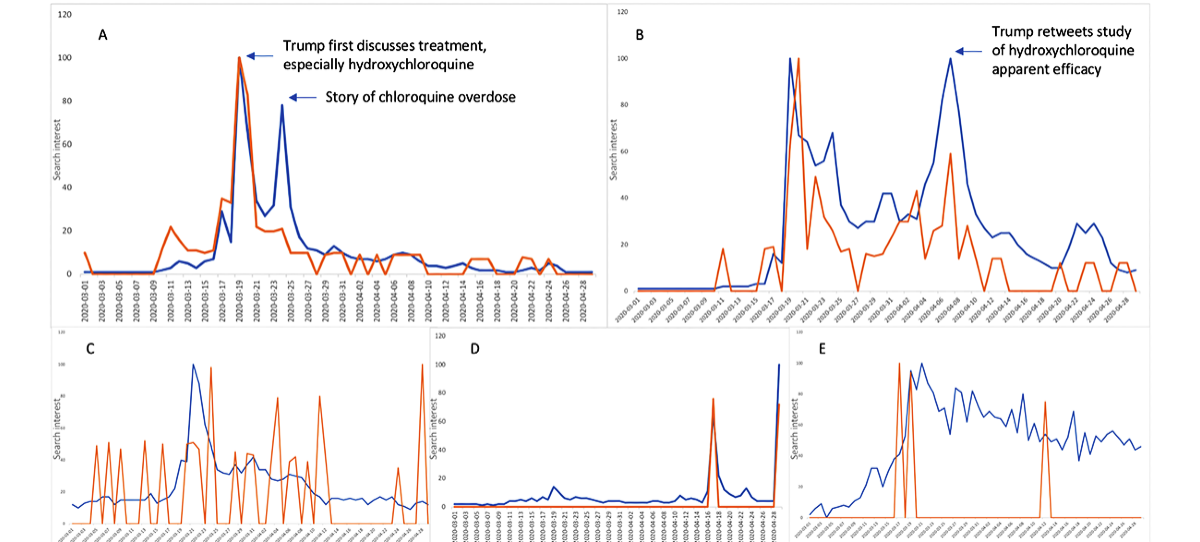
Google searches (blue line) for and purchases (orange line) of keywords. (A) Results for the term "chloroquine." (B) Results for the term "hydroxychloroquine." (C) Results for the term "azithromycin." (D) Results for the term "remdesivir." (E) Results for the term "covid treatment.".

Searches for and purchases (Figure 2A) of chloroquine on Google peaked on March 19, 2020, followed by a second peak on March 23, 2020, after the story of chloroquine-poisoning was made public. Searches for and purchases of hydroxychloroquine followed a similar trend, though there was a second peak on April 4, 2020 when Donald J Trump retweeted a studying discussing the apparent efficacy of hydroxychloroquine. Peaks in COVID-19 treatment searches (Figure 1E) largely coincided with Donald J Trump’s White House briefing on March 19, 2020, with 3 peaks correlating with his tweet history in Table 1 (March 19, March 21, and April 4, 2020).

**Figure 3:**
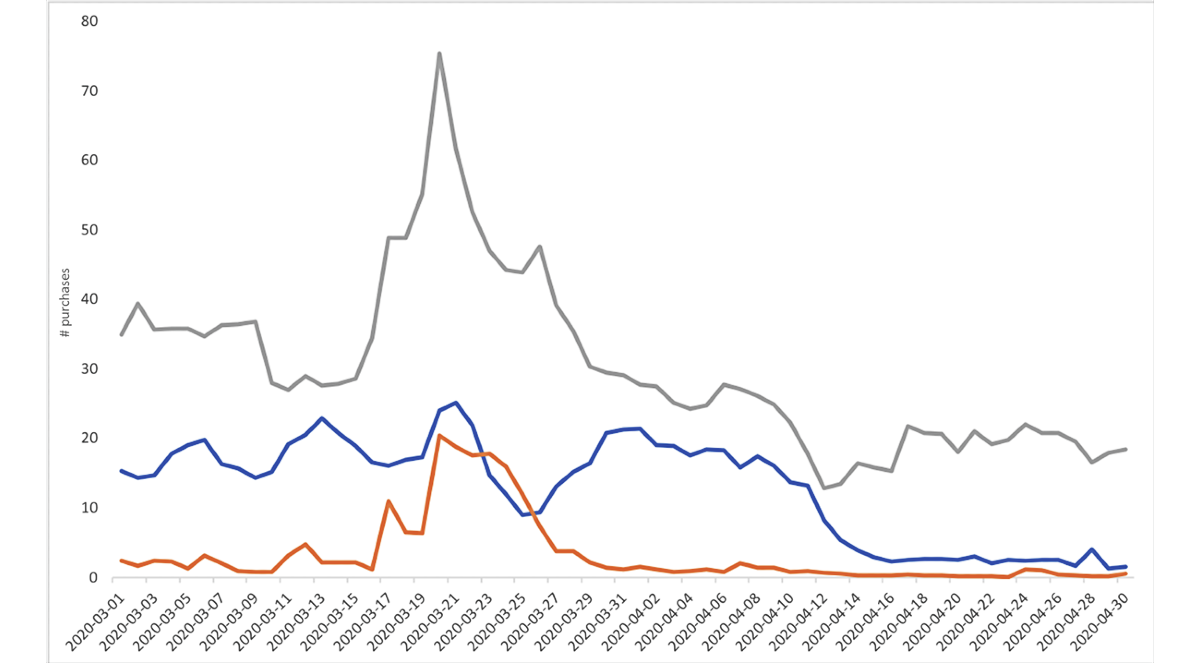
Amazon purchases of alternate therapies. Grey represents purchases of hydroxychloroquine, blue represents purchases of azithromycin, and orange represents purchases of chloroquine.

Our Amazon purchase analysis (Figure 3) showed a peak in purchases for chloroquine, hydroxychloroquine, and azithromycin after the March 19, 2020 White House briefing. This increase in purchases was observed for various forms of these medications, whether it was for purchases of a book about azithromycin [29], which increased by 10 sales; herbal elements claiming to have hydroxychloroquine as an active element [30], which increased by an estimated 50 sales; or texts on alternative chloroquine-containing compounds, such as chloroquine phosphate, which increased by an estimated 30 sales [31]. Secondary peaks for searches on and purchases for hydroxychloroquine were observed after the tweet on April 4, 2020.

## Discussion

This study is the first to show the media landscape of COVID-19 therapies prior to gaining approval or complete disregard from the scientific community. Our results show that there was a substantial increase in purchases and searches for previously unpurchased and unsearched therapies by the general public following the backing of US president Donald J Trump. These increases correlated with his discussions in press conferences and personal social media posts advocating for hydroxychloroquine and chloroquine cures. Conservative outlets provided the most airtime to hydroxychloroquine and chloroquine, and airtime for these therapies only peaked on liberal media outlets after the chloroquine-induced death occurred. Many of the increases in the purchases of these products mirror the increases in searches and airtimes that followed the initial press conference on March 19, 2020, during which Donald J Trump touted these therapies, and the subsequent social media endorsements on Twitter by Donald J Trump. Remdesivir, the treatment with the most evidence from small preliminary studies and largescale testing for COVID-19, had the least coverage time from all stations assessed in this study until just before its emergency approval on May 1, 2020. Donald J Trump has not advocated for its use publicly, except during the initial press conference on March 19, 2020, when all the suggested treatments were outlined.

During unknown medical situations, delicacy is required when determining the best treatments. Previous studies have shown that individuals are susceptible to easy claims and conspiracies without appropriate evidence [32], and once these inauthentic claims are given momentum, they are hard to dissuade [33]. It is for this reason that individuals often seek out influential figures for guidance and knowledge [34]. Providing assurances for unverified claims and treatments is dangerous, given that medications can have numerous side effect profiles. Recent trials have in fact been halted due to their general risk, as was the case with hydroxychloroquine [35]. Additionally, their utility for approved conditions, such as malaria, has been compromised due to limited access and hoarding [36].

Efforts need to be made to prevent further harm. In some instances, this has already occurred. Google has decreased access to links that sell chloroquine, whereas Amazon has removed links to chloroquine phosphate and provided COVID-19–related information on associated links. However, this is only true for the US version of the website. Other domains, such as .ca, still provide easy purchasing for medicine substitutes [37]. Public heath individuals must do more to advocate for safer, evidenced-based approaches. For example, Twitter has recently provided users the ability to flag COVID-19 misinformation and take down posts. This has occurred to Donald J Trump twice after April 30, 2020 for making nonfactual hydroxychloroquine claims [5].

There are limitations in this study. The estimated number of purchases and searches on Google and Amazon were both estimates, as they were provided by external providers rather than the services themselves. However, given the limited data access, these estimates are the best available to third party providers and researchers. Still, ours is first study to examine the purchasing behaviors of individuals and the media landscape of COVID-19 treatments following Donald J Trump’s endorsements. Future studies will examine the searches and purchases of certain key words/items, such as masks, ultraviolet, and disinfectants.

## Abbreviations

BBC: British Broadcasting Corporation
CNN: Cable News Network
C-SPAN: Cable-Satellite Public Affairs Network
MSNBC: Microsoft/National Broadcasting Company
